# Validation of nomogram-revised risk index and comparison with other models for extranodal nasal-type NK/T-cell lymphoma in the modern chemotherapy era: indication for prognostication and clinical decision-making

**DOI:** 10.1038/s41375-020-0791-3

**Published:** 2020-03-09

**Authors:** Si-Ye Chen, Yong Yang, Shu-Nan Qi, Ying Wang, Chen Hu, Xia He, Li-Ling Zhang, Gang Wu, Bao-Lin Qu, Li-Ting Qian, Xiao-Rong Hou, Fu-Quan Zhang, Xue-Ying Qiao, Hua Wang, Gao-Feng Li, Yu-Jing Zhang, Yuan Zhu, Jian-Zhong Cao, Sheng-Min Lan, Jun-Xin Wu, Tao Wu, Su-Yu Zhu, Mei Shi, Li-Ming Xu, Zhi-Yong Yuan, Joachim Yahalom, Richard Tsang, Yu-Qin Song, Jun Zhu, Hang Su, Ye-Xiong Li

**Affiliations:** 1 National Cancer Center/National Clinical Research Center for Cancer/Cancer Hospital, Chinese Academy of Medical Sciences (CAMS) and Peking Union Medical College (PUMC), Beijing, PR China; 2 Chongqing University Cancer Hospital & Chongqing Cancer Hospital, Chongqing, PR China; 3 Division of Biostatistics and Bioinformatics, The Sidney Kimmel Comprehensive Cancer Center, Johns Hopkins University School of Medicine, Baltimore, MD 21205-2013 USA; 4 Jiangsu Cancer Hospital & Jiangsu Institute of Cancer Research, Nanjing, Jiangsu PR China; 5 Union Hospital, Tongji Medical College, Huazhong University of Science and Technology, Wuhan, Hubei PR China; 6 The General Hospital of Chinese People’s Liberation Army, Beijing, PR China; 7 The Affiliated Provincial Hospital of Anhui Medical University, Hefei, Anhui PR China; 8 Peking Union Medical College Hospital, Chinese Academy of Medical Sciences (CAMS) and Peking Union Medical College (PUMC), Beijing, PR China; 9 The Fourth Hospital of Hebei Medical University, Shijiazhuang, PR China; 10 The Second Affiliated Hospital of Nanchang University, Nanchang, PR China; 11 Beijing Hospital, National Geriatric Medical Center, Beijing, PR China; 12 State Key Laboratory of Oncology in South China, Sun Yat-sen University Cancer Center; Collaborative Innovation Center for Cancer Medicine, Guangzhou, PR China; 13 Zhejiang Cancer Hospital, Hangzhou, Zhejiang PR China; 14 Shanxi Cancer Hospital and the Affiliated Cancer Hospital of Shanxi Medical University, Taiyuan, Shanxi PR China; 15 Fujian Provincial Cancer Hospital, Fuzhou, Fujian PR China; 16 Affiliated Hospital of Guizhou Medical University, Guizhou Cancer Hospital, Guiyang, Guizhou PR China; 17 Hunan Cancer Hospital and the Affiliated Cancer Hospital of Xiangya School of Medicine, Changsha, Hunan PR China; 18 Xijing Hospital of Fourth Military Medical University, Xi’an, PR China; 19 Tianjin Medical University Cancer Institute & Hospital, Key Laboratory of Cancer Prevention and Therapy, National Clinical Research Center for Cancer, Tianjin, PR China; 20 Memorial Sloan Kettering Cancer Center, New York, NY USA; 21 Princess Margaret Hospital, University Health Network, University of Toronto, Toronto, ON Canada; 22 Key Laboratory of Carcinogenesis and Translational Research (Ministry of Education), Peking University Cancer Hospital & Institute, Beijing, PR China; 23 The Fifth Medical Center of PLA General Hospital, Beijing, PR China

**Keywords:** T-cell lymphoma, Risk factors

## Abstract

Derived from our original nomogram study by using the risk variables from multivariable analyses in the derivation cohort of 1383 patients with extranodal NK/T-cell lymphoma, nasal-type (ENKTCL) who were mostly treated with anthracycline-based chemotherapy, we propose an easily used nomogram-revised risk index (NRI), validated it and compared with Ann Arbor staging, the International Prognostic Index (IPI), Korean Prognostic Index (KPI), and prognostic index of natural killer lymphoma (PINK) for overall survival (OS) prediction by examining calibration, discrimination, and decision curve analysis in a validation cohort of 1582 patients primarily treated with non-anthracycline-based chemotherapy. The calibration of the NRI showed satisfactory for predicting 3- and 5-year OS in the validation cohort. The Harrell’s C-index and integrated Brier score (IBS) of the NRI for OS prediction demonstrated a better performance than that of the Ann Arbor staging system, IPI, KPI, and PINK. Decision curve analysis of the NRI also showed a superior outcome. The NRI is a promising tool for stratifying patients with ENKTCL into risk groups for designing clinical trials and for selecting appropriate individualized treatment.

## Introduction

Extranodal natural killer/T-cell lymphoma, nasal-type (ENKTCL) is a heterogeneous disease with variable clinical features and prognoses [1–5]. It is endemic in East Asia and South America [1, 2] and accounts for 15–30% of all lymphoma cases in China [6]. ENKTCL is mainly a localized disease with extensive primary tumor invasion (PTI); most patients have stage I/II disease at diagnosis (70–90%), and involvement of distant lymph nodes and/or extranodal sites (stage III/IV, 10–30%) is uncommon [1–5]. In the past decade, the wide use of first-line radiotherapy and non-anthracycline-based chemotherapy has improved ENKTCL treatment and prognosis [7–10]. However, patients are currently managed heterogeneously, primarily depending on the Ann Arbor staging system. Radiotherapy, in particular, plays a curative role in early-stage ENKTCL [7, 8, 11–13], and significantly improves survival even after complete response to asparaginase-based chemotherapy [14]. Nevertheless, despite aggressive chemotherapy, advanced-stage patients generally have poor prognosis [12, 15].

The implementation of an optimal risk classification model for ENKTCL would provide significant advancement in prognostication and would improve outcomes for patients through refined stratification and more relevant information in clinical decision-making. Several models, such as the International Prognostic Index (IPI), Korea Prognostic Index (KPI), and prognostic index of natural killer lymphoma (PINK) have been validated in patients with ENKTCL [15–18]. However, they could not predict prognosis consistently or help physicians tailor initial therapy, or failed to identify risk groups for early-stage patients with favorable prognosis. We previously developed and validated a nomogram for predicting survival for individual patients who were mostly treated with anthracycline-based chemotherapy and conventional radiotherapy [19]. Recently, efforts to improve the model’s discrimination have focused on adding new prognostic factors, regrouping the original prognostic index in various cohorts, or specifically focusing on early-stage patients [12, 20, 21]. None of these models has undergone comprehensive evaluation to provide further evidence for efficacy and general applicability. Moreover, the certainty of existing models requires further verification in the era of modern treatment. Based on our previous study [19], we propose here an easily used nomogram-revised risk index (NRI), and validate its predictive value for all patients with ENKTCL, particularly early-stage patients. We also compare the relative accuracy of its predictive performance and usefulness of clinical decision-making with the commonly used models in the modern chemotherapy era.

## Patients and methods

### Eligibility criteria and study population

As described previously in detail [22], the China Lymphoma Collaborative Group (CLCG) comprises a group of nationwide institutions in China. ENKTCL data collection started in January 2011, and was updated in March 2016. We censored the analysis to July 2017. The current CLCG database included 3046 consecutive patients treated in 20 China institutions between 2000 and 2016. In addition, 51 patients with newly diagnosed ENKTCL who received non-anthracycline-based chemotherapy at the Memorial Sloan Kettering Cancer Center, USA and Princess Margaret Cancer Center, Canada between 2000 and 2016 were enrolled into the CLCG database. Our institutional ethics review board approved the study and waived the need for informed consent, as patients had been de-identified in the dataset. Given the wide use of new chemotherapy regimens and modern radiotherapy in the past decade [21, 22], the eligibility criteria for this validation study included: (1) patients who had received initial treatment between 2008 and 2016; (2) patients who had received non-anthracycline-based chemotherapy with or without radiotherapy, or radiotherapy alone as the first-line treatment. The exclusion criteria were: (1) patients treated before 2008; (2) anthracycline-based or unknown chemotherapy regimens; (3) dataset from the study of our original nomogram. The pretreatment evaluations and definition of PTI have been described previously [22].

### NRI risk classification

In our original nomogram model [19], the risk variables from multivariable Cox model regression analysis included age >60 years, Eastern Cooperative Oncology Group (ECOG) score ≥2, elevated lactate dehydrogenase (LDH), PTI, stage II, and stage III/IV. According to these factors and corresponding regression coefficients (or equivalently, nomogram scores), we proposed an easily applicable NRI for clinical convenience and easy memorization. The process of deriving the NRI is similar to other commonly used models of ENKTCL or diffuse large B-cell lymphoma [14–19]. Comparisons of different models and the hazard radio (HR) of variables in the original IPI, KPI, PINK, and NRI models were presented in the Table 1 [15, 16, 18, 19]. The HRs of variables in the original nomogram study were between 1 and 2, except for stage III/IV disease (HR = 3.6) [19]. Accordingly, the adopted weights of single NRI components were as follows: 1 point each for the risk factor age >60 years, ECOG score ≥2, elevated LDH, PTI, or stage II; 2 points for stage III/IV disease. The resulting distribution of the NRI is similar to the original nomogram (Supplementary Fig. 1A, B). Patients were stratified into one of five risk groups by combining the indices of these parameters (low, 0; intermediate low, 1; intermediate high, 2; high, 3; very high, ≥4). Excluding stage III/IV disease, the NRI stratified early-stage patients into one of four risk groups (low, 0; intermediate low, 1; intermediate high, 2; high, ≥3).

**Table 1:** Comparison of different models and the HR of variables in the original IPI, KPI, PINK and NRI models.

Model and definition (total point)	Variable	HR^a^	Point	Nomogram score [19]
NRI				
Low risk (0)	Age (>60 years vs. ≤60 years)	1.35	1	24
Intermediate low risk (1)	Ann Arbor stage			
Intermediate high risk (2)	II (II vs. I)	1.86	1	48
High risk (3)^b^	III–IV (III/IV vs. I)	3.60	2	100
Very high risk (≥4)	ECOG score (≥2 vs. 0–1)	1.84	1	48
	Elevated LDH (yes vs. no)	1.33	1	22
	PTI (yes vs. no)	1.78	1	45
IPI [16]				
Low (0–1)	Age (>60 years vs. ≤60 years)	1.96	1	
Intermediate low (2)	Ann Arbor stage (III/IV vs. I/II)	1.47	1	
Intermediate high (3)	ECOG score (≥2 vs. 0–1)	1.80	1	
High (≥4)	Elevated LDH (yes vs. no)	1.85	1	
	Distant extranodal involvement (≥2 vs. 0–1)	1.48	1	
KPI [18]				
Group 1 (0)	Ann Arbor stage (III/IV vs. I/II)	2.37	1	
Group 2 (1)	Elevated LDH (yes vs. no)	2.28	1	
Group 3 (2)	B symptoms (yes vs. no)	2.20	1	
Group 4 (≥3)	Regional lymph node (yes vs. no)	1.55	1	
PINK [15]				
Low risk (0)	Age (>60 years vs. ≤60 years)	2.17	1	
Intermediate risk (1)	Ann Arbor stage (III/IV vs. I/II)	2.57	1	
High risk (≥2)	Distant lymph node involvement (yes vs. no)	1.73	1	
	Nonnasal disease (yes vs. no)	1.94	1	

*HR* hazard ratio, *IPI* International Prognostic Index, *KPI* Korean Prognostic Index, *PINK* prognostic index of natural killer lymphoma, *NRI* nomogram-revised risk index, *ECOG* Eastern Cooperative Oncology Group, *LDH* lactate dehydrogenase, *PTI* primary tumor invasion.

^a^Data derived from the original publication.

^b^High-risk group was defined as NRI ≥ 3 for early-stage patients.

### Endpoints and statistics

The primary endpoint was overall survival (OS), defined as the time from the start of treatment to death from any cause or to the last follow-up. Progression-free survival (PFS) was defined as the time from the start of treatment to disease progression, relapse, or death. Survivals were estimated by the Kaplan–Meier method and compared with a log-rank test.

Predicted survival probabilities from the derivation cohort by applying the NRI to the baseline survival estimate at the individual level in the validation cohort, and averaging across each risk group [23]. Then the predicted mean survival is compared with the Kaplan–Meier survival in the validation cohort. The time-dependent receiver operating characteristic (tROC) and corresponding area under curve (tAUC) and Harrell’s C-index were used to evaluate model discrimination [24, 25]. The time-dependent ROC and AUC compute the sensitivity (true-positive rate) against one minus specificity (false-positive rate) for consecutive cutoffs for the predicted risk and over time. A high Harrell’s C- index suggests high discriminatory value for censored data where 0.5 represents non-informative discrimination. In addition, the cumulative prediction errors or integrated Brier score (IBS) was used to evaluate prediction accuracy over time, with IPCW (inverse of the probability of censoring weights) to account for censoring and cross-validation used to avoid overfitting [26]. Decision curve analysis was used to determine whether the models could be considered useful tools for clinical decision-making by comparing the net benefits at any threshold probability [27]. Statistical analyses were performed using IBM SPSS Statistics, Version 25.0, and packages of “survival”, “rms”, “timeROC”, “pec”, “dynpred”, and “rmda” in R version 3.4.4 (http://www.r-project.org/).

## Results

### Clinical features, treatment and survival

A CONSORT diagram describing the cohort selection is outlined (Supplementary Fig. 2). The derivation cohort included 1383 ENKTCL patients mostly treated with anthracycline-based chemotherapy [19], whereas the validation cohort included 1582 patients treated with non-anthracycline-based chemotherapy for independent validation and comparison. Table 2 lists the clinical characteristics in the whole validation cohort and in the early-stage patients. The male-to-female ratio was 2.4:1; the median age was 44 years. PTI was present in 55.2% of patients. The majority of patients were aged ≤60 years (84.5%), had stage I/II disease (86.5%), and had good performance status (93.5%), normal LDH (73.6%), and tumor originating from the upper aerodigestive tract (UADT, 92.5%). Only a few patients had involvement of the distant lymph nodes (5.9%), extranodal sites (10.4%), or multiple extranodal sites (3.4%). Regional lymph node involvement was present in 59.1% of stage III/IV patients (*n* = 127).

**Table 2:** Baseline of clinical features and distribution of risk groups. According to different prognostic models for all patients and early-stage patients in the validation cohort.

Characteristic	All	Early stage
	No. (%)	No. (%)
Total number	1582 (100)	1367 (86.4)
Sex		
Male	1110 (70.2)	956 (69.9)
Female	472 (29.8)	411 (30.1)
Age (years)		
≤60	1336 (84.5)	1149 (84.1)
>60	246 (15.5)	218 (15.9)
Primary site		
UADT (nasal)	1464 (92.5)	1325 (96.9)
Extra-UADT (nonnasal)	118 (7.5)	42 (3.1)
Regional lymph nodes		
Yes	604 (38.2)	477 (34.9)
No	978 (61.8)	890 (65.1)
Distant lymph nodes		
Yes	91 (5.8)	NA
No	1491 (94.2)	1367 (100)
PTI		
Yes	873 (55.2)	732 (53.5)
No	709 (44.8)	635 (46.5)
B symptoms		
Yes	628 (39.7)	516 (37.7)
No	954 (60.3)	851 (62.3)
Elevated LDH		
Yes	418 (26.4)	308 (22.5)
No	1164 (73.6)	1059 (77.5)
ECOG score		
0–1	1479 (93.5)	1314 (96.1)
≥2	103 (6.5)	53 (3.9)
Distant extranodal organs		
0	1417 (89.6)	1367 (100)
1	111 (7.0)	NA
≥2	54 (3.4)	NA
Ann Arbor stage		
I	890 (56.3)	890 (56.3)
II	477 (30.2)	477 (30.2)
III	50 (3.2)	NA
IV	165 (10.4)	NA
IPI		
Low (0–1)	1354 (85.6)	1289 (94.3)
Intermediate low (2)	145 (9.2)	69 (5.0)
Intermediate high (3)	65 (4.1)	9 (0.7)
High (≥4)	18 (1.1)	0 (0)
KPI		
Group 1 (0)	490 (31.0)	490 (35.8)
Group 2 (1)	563 (35.6)	532 (38.9)
Group 3 (2)	330 (20.8)	266 (19.5)
Group 4 (≥3)	199 (12.6)	79 (5.8)
PINK		
Low risk (0)	1088 (68.8)	1088 (79.6)
Intermediate risk (1)	334 (21.1)	260 (19.0)
High risk (≥2)	160 (10.1)	19 (1.4)
NRI		
Low risk (0)	352 (22.3)	352 (25.7)
Intermediate low risk (1)	447 (28.3)	447 (32.7)
Intermediate high risk (2)	423 (26.7)	395 (28.9)
High risk (3)^a^	223 (14.1)	173 (12.7)
Very high risk (≥4)	137 (8.7)	NA

*UADT* upper aerodigestive tract, *PTI* primary tumor invasion, *LDH* lactate dehydrogenase, *ECOG* Eastern Cooperative Oncology Group, *NA* not available, *IPI* International Prognostic Index, *KPI* Korean Prognostic Index, *PINK* prognostic index of natural killer lymphoma, *NRI* Nomogram-revised risk index.

^a^High-risk group was defined as NRI ≥ 3 for early-stage patients.

The patients had received chemotherapy alone (*n* = 295, 18.6%), radiotherapy alone (*n* = 252, 15.9%), or combined modality therapy (*n* = 1035, 65.4%) in our validation cohort. Of the patients who had received non-anthracycline–based chemotherapy, the majority (*n* = 1176, 88.4%) had received asparaginase- or gemcitabine-containing regimens, and the remaining 154 (11.6%) patients had received platinum- or etoposide-containing regimens. Of early-stage patients who had received definitive radiotherapy, most had received extended involved-site intensity-modulated radiotherapy (IMRT) or three-dimensional conformal radiotherapy (88.4%) and a ≥50 Gy dose (88.6%).

**Fig. 1:**
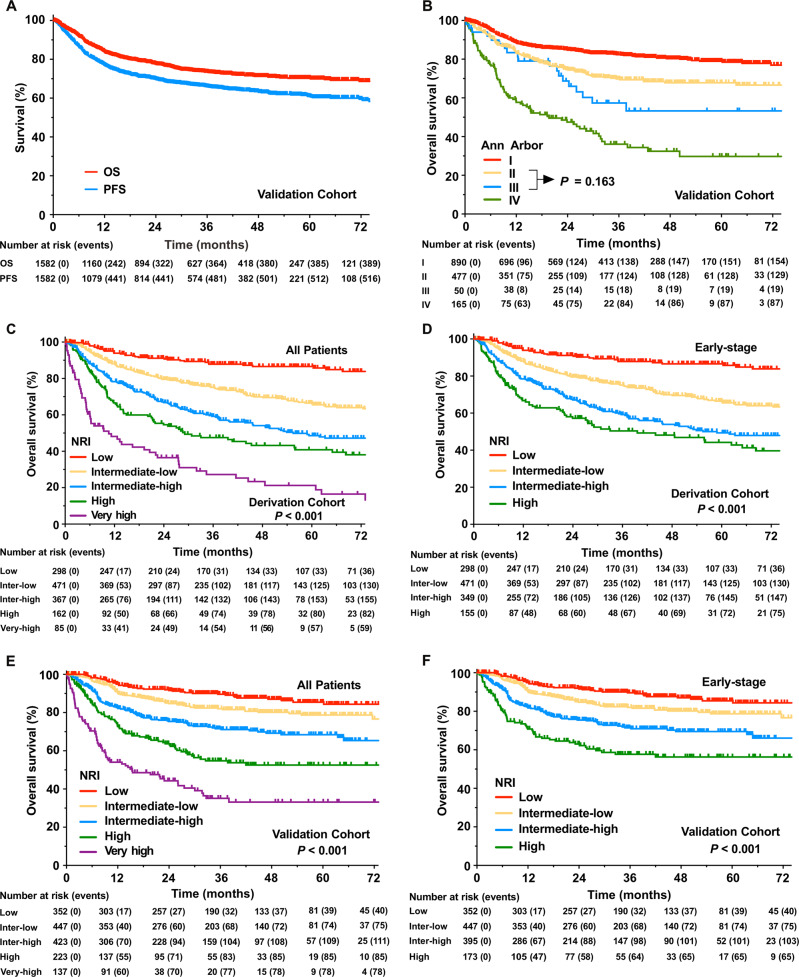
Survival curves. Overall survival (OS) and progression-free survival (PFS) in the whole validation cohort (**a**). OS stratified by the Ann Arbor staging system in the validation cohort (**b**). OS stratified by the nomogram-revised risk index (NRI) for all patients (**c**) and early-stage patients (**d**) in the derivation cohort, and for all patients (**e**) and early-stage patients (**f**) in the validation cohort.

With a median follow-up time of 37 months for surviving patients in the validation cohort, the 5-year OS and PFS were 70.2% and 60.9% in all patients (Fig. 1a), respectively, 75.5% and 65.6% in patients with early-stage disease, respectively, and 35.9% and 28.0% in patients with advanced-stage disease, respectively. The 5-year OS was 78.9%, 67.8%, 53.3%, and 29.7% for Ann Arbor stage I, II, III, and IV, respectively (Fig. 1b).

### Validation of NRI

Comparison of the histograms of the NRI and nomogram score showed minimal differences in the derivation (Supplementary Fig. 1A) and validation cohorts (Supplementary Fig. 1B). Similar cumulative distributions of the nomogram score (Supplementary Fig. 1C) and NRI score (Supplementary Fig. 1D) were also observed in the derivation and validation datasets. In the derivation cohort, stratifying the whole population according to the NRI categories classified 298 (21.5%) patients as low risk, 471 (34.1%) as intermediate low risk, 367 (26.5%) as intermediate high risk, 162 (11.7%) as high risk, and 85 (6.2%) as very high risk. Similarly, in the validation cohort, 352 (22.3%) patients were classified as low risk, 447 (28.3%) as intermediate low risk, 423 (26.7%) as intermediate high risk, 223 (14.1%) as high risk, and 137 (8.7%) as very high risk. The 5-year OS in the low, intermediate low, intermediate high, high, and very high risk categories was 86.6%, 66.4%, 48.6%, 40.8%, and 21.2% in the derivation cohort (*P* < 0.001, Fig. 1c), and 85.4, 78.7%, 68.4%, 52.5%, and 33.2% in the validation cohort, respectively (*P* < 0.001, Fig. 1e).

Calibrations of 3-year and 5-year OS by the NRI were presented in the Table 3. Predicted 3- and 5-year OS in the validation cohort were calculated from the derivation cohort by applying the NRI to the baseline survival estimate at the individual level, and averaging across each risk group. There was minimal difference between the observed OS (S (t)) by Kaplan–Meier method and the predicted OS ($\overline {\mathrm{S}}$ (t)) in all risk groups at 3 years (median follow-up time), and in the low or intermediate risk groups at 5 years. There was an apparent difference between the observed and predicted 5-year OS in high and very high risk groups, likely due to the small sample size and improved survival with non-anthracycline-based chemotherapy in such patients from the validation cohort [22]. Furthermore, the calibration curve for the probability of 5-year OS showed good correlation between the actual observation and the NRI prediction in the whole derivation cohort (Supplementary Fig. 3A) and the whole validation cohort (Supplementary Fig. 3C).

**Table 3:** Calibration of 3-year and 5-year OS by the nomogram-revised risk index.

NRI defined	*t* (year)	Estimation by Kaplan–Meier method								Predicted OS in validation cohort
		Observed OS in derivation cohort				Observed OS in validation cohort				
Risk group		No.	Ev.	*S* (*t*)	SE	No.	Ev.	*S* (*t*)	SE	$\overline {\mathit{S}}$(*t*)
Low risk	3	298	39	0.878	0.021	352	40	0.896	0.018	0.892
	5			0.866	0.022			0.854	0.023	0.859
Intermediate low risk	3	471	140	0.751	0.022	447	75	0.820	0.020	0.823
	5			0.664	0.026			0.787	0.024	0.767
Intermediate high risk	3	367	161	0.592	0.028	423	113	0.716	0.024	0.701
	5			0.486	0.031			0.684	0.027	0.628
High risk	3	162	83	0.475	0.043	223	85	0.548	0.038	0.539
	5			0.408	0.045			0.525	0.040	0.442
Very high risk	3	85	63	0.272	0.055	137	79	0.351	0.048	0.349
	5			0.212	0.053			0.332	0.049	0.242

*OS* overall survival, *NRI* nomogram-revised risk index, *t* follow-up time, *Ev*. number of events, *S* (*t*) observed overall survival probabilities at the follow-up time, *SE* standard error, $\overline {\mathit{S}}$ (*t*) predictive overall survival probabilities from the derivation cohort by applying the nomogram-revised risk index to the baseline survival estimate at the individual level in the validation cohort, and averaging across each risk group.

We specifically analyzed the performance of the NRI for early-stage patients. The 5-year OS in the low, intermediate low, intermediate high, and high risk groups was 86.6%, 66.4%, 49.3%, and 44.2% in the derivation cohort (*P* < 0.001, Fig. 1d), and 85.4%, 78.7%, 69.5%, and 56.3% in the validation cohort, respectively (*P* < 0.001, Fig. 1f). The calibration curves presented an excellent agreement between the NRI prediction and actual observation for 5-year OS in the derivation cohort (Supplementary Fig. 3B) and validation cohort (Supplementary Fig. 3D). The results suggest that the NRI can stratify all patients or early-stage patients into four or five risk groups with different outcomes not only in the anthracycline-based chemotherapy era but also in the non-anthracycline-based chemotherapy era.

### Comparison of OS between models

**Fig. 2:**
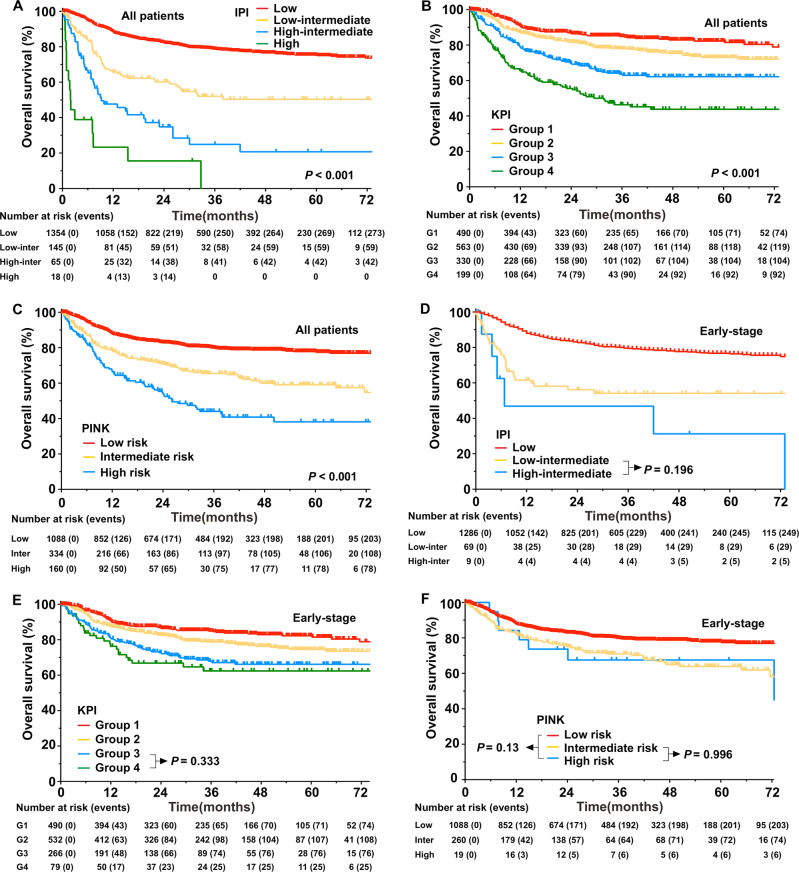
Overall survival (OS) stratified by prognostic models in the validation cohort. **a** International prognostic index (IPI), **b** Korean Prognostic Index (KPI), and **c** prognostic index of natural killer lymphoma (PINK) for all patients. **d** IPI, **e** KPI, and **f** PINK for early-stage patients.

We compared the NRI with other models and the Ann Arbor staging for the entire and early-stage in the validation cohorts. The prognostic value varied between the models and across cohorts. The IPI, KPI, and PINK presented a good level of OS prediction with a risk-adopted classification for all patients (all, *P* < 0.001, Fig. 2a–c). However, the IPI and PINK classified most patients (~90%) as low and intermediate risk (Table 2). The IPI and KPI could not differentiate intermediate and high-risk groups in the early-stage patients (Fig. 2d, e), whereas the PINK could not discriminate at-risk patients in the early-stage cohort (Fig. 2f).

**Fig. 3:**
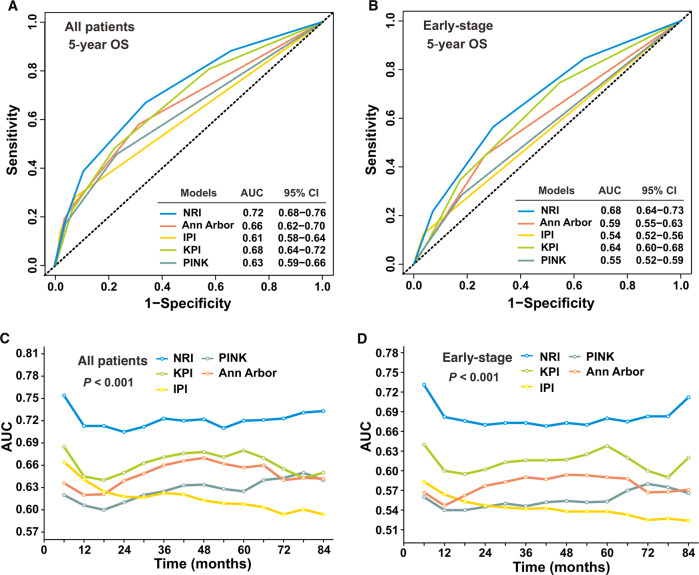
Validation and comparison of the nomogram-revised risk index (NRI). The NRI was validated and compared with the international prognostic index (IPI), Ann Arbor staging, Korean Prognostic Index (KPI), and prognostic index of natural killer lymphoma (PINK) models by the area under the curve (AUC) in the validation cohort. The AUC for predicting 5-year overall survival (OS) for all-stage (**a**) and early-stage (**b**) patients. The time-dependent AUC between 6 and 84 months for all-stage (**c**) and early-stage (**d**) patients.

Compared with the other models, the NRI had better levels of accuracy for predicting OS in the validation cohort (Table 4). The NRI AUC for predicting the 5-year OS (0.72, 95% CI: 0.68–0.76) for all patients was significantly higher than that of the KPI (0.68, 95% CI: 0.64–0.72), PINK (0.63, 95% CI: 0.59–0.66), IPI (0.61, 95% CI: 0.58–0.64), and Ann Arbor staging (0.66, 95% CI: 0.62–0.70; *P* < 0.01, Fig. 3a). Similarly, early-stage patients had significantly lower AUC of the KPI (0.64, 95% CI: 0.60–0.68), PINK (0.55, 95% CI: 0.52–0.59), IPI (0.54,95% CI: 0.52–0.56), and Ann Arbor staging (0.59, 95% CI: 0.55–0.63) than that of the NRI (0.68, 95% CI: 0.64–0.73; *P* < 0.005, Fig. 3b). Furthermore, the NRI tAUC between 6 and 84 months was consistently higher than that of the other models in the entire validation cohort and early-stage patients (all, *P* < 0.001; Fig. 3c, d). The Harrell’s C-index of the NRI (0.70, 95% CI: 0.67–0.73; 0.66, 95% CI: 0.63–0.69) was consistently higher than that of the KPI (0.64, 95% CI: 0.62–0.67; 0.60, 95% CI: 0.56–0.63), Ann Arbor staging (0.63, 95% CI: 0.60–0.66; 0.56, 95% CI: 0.54–0.59), IPI (0.62, 95% CI: 0.59–0.64; 0.55, 95% CI: 0.53–0.57), and PINK (0.61, 95% CI: 0.59–0.64; 0.55, 95% CI: 0.52–0.57) in the entire cohort and early-stage cohort.

**Table 4:** The 5-year overall survival according to risk group as defined by prognostic models for all patients and early-stage patients in the validation cohort.

Risk group	All		Early stage	
	% (95% CI)	*P*	% (95% CI)	*P*
NRI		<0.001		<0.001
Low	85.4 (80.9–89.9)		85.4 (80.9–89.9)	
Intermediate low	78.7 (74.0–83.4)		78.7 (74.0–83.4)	
Intermediate high	68.4 (63.1–73.7)		69.5 (64.2–74.8)	
High	52.5 (44.7–60.3)		56.3 (47.9–64.7)	
Very high	33.2 (23.6–42.8)		–	
Ann Arbor stage		<0.001		<0.001
I	78.9 (75.6–82.2)		78.9 (75.6–82.2)	
II	67.8 (62.9–72.7)		67.8 (62.9–72.7)	
III	53.3 (37.0–69.6)		–	
IV	29.7 (19.5–39.9)		–	
IPI		<0.001		<0.001
0–1	75.2 (72.4–77.9)		76.5 (73.7–79.2)	
2	50.3 (40.7–60.0)		54.1 (41.5–66.6)	
3	20.7 (7.4–34.0)		31.3 (1.3–66.2)	
4–5	0		–	
KPI		<0.001		<0.001
Group 1	82.3 (78.4–86.2)		82.3 (78.4–86.2)	
Group 2	73.0 (68.5–77.5)		74.5 (70.0–79.0)	
Group 3	62.2 (56.1–68.3)		66.1 (59.6–72.6)	
Group 4	43.8 (35.6–52.0)		62.4 (50.4–74.4)	
PINK		<0.001		<0.001
Low risk	77.8 (74.9–80.7)		77.8 (74.9–80.7)	
Intermediate risk	59.1 (52.6–65.6)		63.9 (56.6–71.2)	
High risk	38.1 (28.1–48.1)		67.5 (45.9–89.1)	

*NRI* nomogram-revised risk index, *IPI* International Prognostic Index, *KPI* Korean Prognostic Index, *PINK* prognostic index of natural killer lymphoma.

The performance of each model was also assessed by calculating prediction error over time in the entire and early-stage cohorts. The NRI IBS (0.143) of the 5-year OS for all patients was lower than that of the IPI (0.148), KPI (0.152), PINK (0.154), and Ann Arbor staging (0.149). Similarly, early-stage patients had higher IBS of the IPI (0.141), KPI (0.140), PINK (0.143), and Ann Arbor staging (0.141) than that of NRI (0.135). The corresponding prediction error curves of all models in the entire cohort and early-stage cohort were shown in Supplementary Fig. 4A, B. The results suggest that the NRI is a more accurate and useful tool for stratifying and discriminating OS for all patients and early-stage patients in the modern treatment era.

### Comparison of clinical decision-making with models

**Fig. 4:**
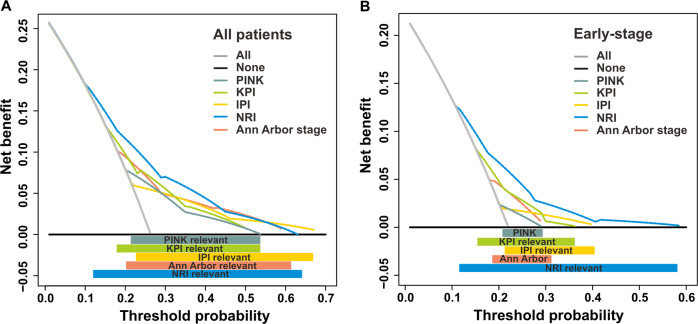
Time-dependent decision curve analysis. Time-dependent decision curve analysis of risk models predicting the 3-year mortality by any cause for all-stage (**a**) and early-stage (**b**) patients in the validation cohort. The threshold probability represented the 3-year risk of mortality by any cause based on each prognostic model for recommending clinical intervention. The threshold defines the weight *w* for false-positive (FP, treat while patient survived) vs. true-positive (TP, treat a patient who died) classifications. The net benefit (NB) balanced the risk of real 3-year mortality with the potential harms of unnecessary intervention (including decision of treatment, work-up, or follow-up) for false prediction and was calculated as the true-positive rate minus the weighted false-positive rate. The clinical usefulness of a prediction model can be summarized as: NB = (TP − *w* FP)/N, where *N* is the total number of patients. Solid black line: Assume no patients need receive clinical intervention (no patients died), net benefit is zero (no true-positive and no false-positive classification). Gray line: Assume all patients need receive clinical intervention (all died). Dotted color lines: Patients received clinical intervention if predictions exceeded a threshold, with 3-year mortality risk predictions based on different prognostic models. In general, the prognostic model with the highest net benefit at any threshold is deemed to have the highest clinical application value.

We used decision curve analysis to evaluate whether the models can guide treatment in the validation cohort. In the whole validation cohort, the NRI had better utility for clinical decision-making than the other models, with a risk probability of 0.11–0.64 (Fig. 4a). The NRI had a larger threshold probability range than the IPI (0.22–0.68), KPI (0.16–0.55), PINK (0.21–0.55), and Ann Arbor staging (0.20–0.60). Moreover, the NRI had the highest net benefit at the threshold probability between 0.11 and 0.42. For early-stage patients, the NRI also obtained the highest net benefit with the widest threshold probability range (0.11–0.55) compared with the other models (Fig. 4b), i.e., the IPI (0.21–0.40), KPI (0.16–0.38), PINK (0.21–0.29), and Ann Arbor staging (0.18–0.30). This result indicates that the NRI model is beneficial for clinical decision-making.

## Discussion

Despite several proposed ENKTCL classification models based on clinical data, there has been no systematic evaluation of these models to date. Using a large multicenter cohort of patients with ENKTCL from the CLCG and North American databases, we verified that a ready-to-use NRI, derived from an original visual nomogram [19], provides better prediction of OS with a high degree of concordance under current treatment strategies, and appears to outperform other commonly used models. More importantly, the NRI is the first risk index specifically designed for predicting survival for early-stage patients who require radiotherapy. Comparison of the models’ performance showed that the NRI was superior to the IPI, KPI, PINK, and Ann Arbor staging in terms of discrimination, risk stratification, predictive accuracy, and clinical decision guidance in the entire cohort and in the early-stage cohort. The remarkable advantage and simplicity of the NRI will accelerate its utility in prospective trials and eventually in clinical routines.

Optimization of risk stratification is important for facilitating prognoses and treatment decisions in a variety of lymphomas [15–18, 28–30]. The NRI is based on a large cohort of patients with ENKTCL primarily treated with non-anthracycline-based chemotherapy and IMRT, representing the mainstay of current clinical practice. We demonstrate that the NRI is easy to use, includes only the most relevant patient and disease features, can accurately distinguish between risk groups of patients with different outcomes, and has superior application in multidisciplinary team decision-making compared with the other models. However, the clinical utility of the IPI, KPI, and PINK for ENKTCL is limited by the use of outdated chemotherapy regimens and inappropriate radiation techniques, lack of external validation, or the lack of segregation of early-stage patients into risk groups [15–18]. The NRI incorporates readily available clinical variables reflecting tumor load (stage, LDH, PTI), invasive potential (stage, PTI), and the patient’s ability to tolerate treatment (age, ECOG score). Unlike the other models and Ann Arbor staging, the NRI includes PTI as a novel independent predictor of survival of ENKTCL, particularly in stage I disease [22]. As a common, exclusive clinical feature of ENKTCL [22, 31], PTI predicting survival is in line with the idea that heavy primary tumor load indicates aggressive disease with a greater probability of progression or relapse [7, 8, 19, 32]. Stage II disease or regional lymph node involvement is another important risk-stratified factor in the NRI and KPI [18, 19], but is overlooked in the PINK and IPI [15, 16]. Our results suggest that models that include localized disease-related risk factors, such as stage II and PTI in the NRI, and regional lymph node involvement in the KPI [18], have better capability for predicting OS for patients with ENKTCL. Consistent with our previous studies [22, 33, 34], integrating PTI and stage II disease into the NRI enables the identification of several discrete risk groups and provides an opportunity for improved treatment allocation. The decreased ability of the other models to discriminate between risk groups can be partially explained by the lack of robust prognostic factors for early-stage patients with favorable prognoses (IPI, PINK), and the overlap between prognostic factors, such as regional lymph node and stage II disease (KPI), and distant lymph node and stage III/IV disease (PINK).

Treatment strategies differ notably between early-stage and advanced-stage ENKTCL. Radiotherapy is the backbone of curative intent for localized disease [7, 8, 11], but not for disseminated disease [35]. Improved locoregional control by radiotherapy is associated with prolonged OS and PFS in early-stage ENKTCL [36]. However, only systematic chemotherapy has the potential to cure patients with advanced or disseminated disease [9, 14, 35]. Due to the heterogeneity of the disease and variations in the combination and intensity of the first-line treatment for early-stage patients (single modality with radiotherapy or chemotherapy, different sequences of radiotherapy and chemotherapy, various chemotherapy regimens of different intensities) [7, 8, 12, 21], it is important to improve risk stratification models, especially for such a large cohort. In the present study, despite improved survival (5-year OS, 75.5%) with current treatment strategies, the 5-year OS rates for early-stage patients varied substantially from 56.3 to 85.4% when stratified by the NRI into risk groups. Consistently [8, 19], the NRI model identified a low-risk subgroup with favorable prognosis (5-year OS, 85–90%) in ~25% of early-stage patients. As observed in recent studies [8, 22, 33, 34], low-risk early-stage patients could be considered for chemotherapy and surveillance reduction in the radiotherapy setting. Furthermore, the NRI was a useful risk-stratified index for better selection of patients who may benefit from additional modern chemotherapy and radiotherapy [8, 13, 22, 33], planning customized follow-up schedules, and tailoring patient counseling based on risk-dependent conditional survival and failure hazard [33, 34]. These findings highlight the unique effect and importance of the NRI in designing prospective trials for early-stage ENKTCL. In contrast, although treated with more effective non-anthracycline-based regimens, patients with stage III/IV disease had extremely poor outcomes (5-year OS, <40%), similar to the high- or very high-risk groups [12, 15]. Consequently, advanced-stage disease is the most important prognostic factor, as consistently identified in all four models [15, 16, 18, 19]. High mortality in patients with advanced-stage disease or at high-risk reflects inherent resistance to chemotherapy, indicating the necessity of intensified chemotherapy or innovative systematic therapy [37].

Although we validated and compared the accuracy of the NRI model using systematic and effective methods, the study has some limitations. First, genomic classifiers were not used, and therefore could not be incorporated into the NRI. However, the NRI may be a promising backbone for more comprehensive risk models integrating molecular and biological markers in the future [38]. Second, despite the wide enrollment of more recently treated patients with ENKTCL from a real-world, international cooperative database, the majority of patients came from an endemic area (China). More work is required to determine whether the NRI model can be applied to patients from non-endemic areas.

In summary, the NRI significantly improves prognostication with respect to the capability for discrimination and the effectiveness of clinical decision-making, and is particularly useful for early-stage patients in the era of modern chemotherapy and radiotherapy. This study provides the basis of prognostic stratification for designing prospective trials of risk-adapted therapies and surveillance strategies.

## Supplementary information

Supplementary Figure legends

Supplementary Figure 1A

Supplementary Figure 1B

Supplementary Figure 1C

Supplementary Figure 1D

Supplementary Figure 2

Supplementary Figure 3A

Supplementary Figure 3B

Supplementary Figure 3C

Supplementary Figure 3D

Supplementary Figure 4A

Supplementary Figure 4B
