# Supplementary material for: Validation of nomogram-revised risk index and comparison with other models for extranodal nasal-type NK/T-cell lymphoma in the modern chemotherapy era: indication for prognostication and clinical decision-making
Source: Leukemia. 2020 Mar 9;35(1):130–42. doi: 10.1038/s41375-020-0791-3 (PMC7787971; doi:10.1038/s41375-020-0791-3)
Supplement: Supplementary file 1 — Supplementary Figure legends [file 41375_2020_791_MOESM1_ESM.docx]

**Validation of Nomogram-Revised Risk Index and Comparison with Other Models for Extranodal Nasal-type NK/T-cell Lymphoma in the Modern Chemotherapy Era: Indication for Prognostication and Clinical Decision-making**

**Supplementary Figure Legends**

**Supplementary Figure 1. Distribution of the original nomogram and nomogram-revised risk index (NRI).** The histograms of NRI and the nomogram groups in the derivation cohort of the nomogram study (A) and the external validation cohort of present study (B). Cumulative distribution function (CDF) of nomogram (C) and NRI score (D) in the derivation cohort and validation cohort.

**Supplementary Figure 2. CONSORT diagram.**

**Supplementary Figure 3. Calibration curves.** The calibration curves for predicting 5-year overall survival (OS) in the derivation cohort (A, B) and the validation cohort (C, D). NRI-predicted OS is plotted on the x-axis; actual OS is plotted on the y-axis. A plot along the 45-degree line would indicate a perfect calibration model in which the predicted probabilities are identical to the actual outcomes.

**Supplementary Figure 4. Prediction error curves.** Prediction error curves of all models for all-stage (A) and early-stage (B) patients in the validation cohort.
