# Supplementary figures and images for: Validation of nomogram-revised risk index and comparison with other models for extranodal nasal-type NK/T-cell lymphoma in the modern chemotherapy era: indication for prognostication and clinical decision-making
Source: Leukemia. 2020 Mar 9;35(1):130–42. doi: 10.1038/s41375-020-0791-3 (PMC7787971; doi:10.1038/s41375-020-0791-3)

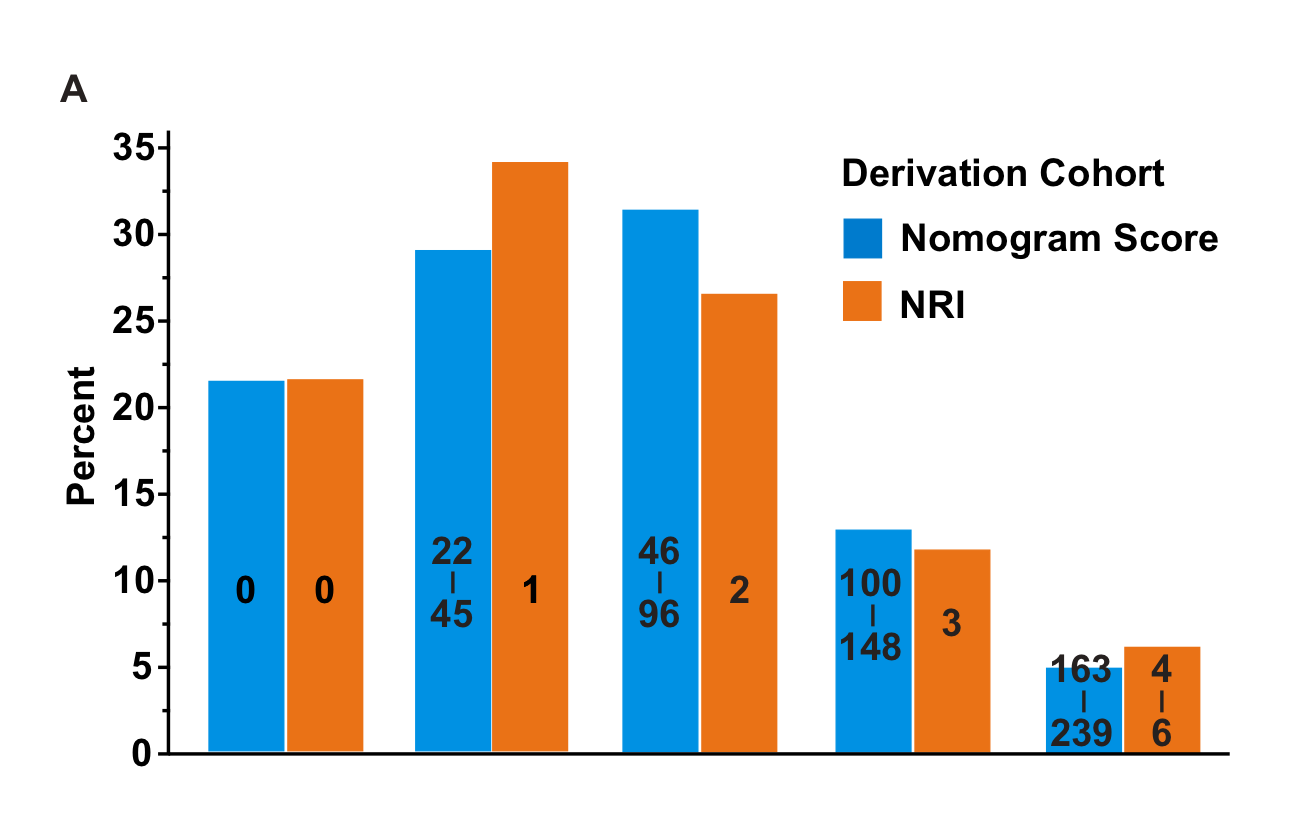

Supplement: Supplementary file 2 — Supplementary Figure 1A [file 41375_2020_791_MOESM2_ESM.tif]

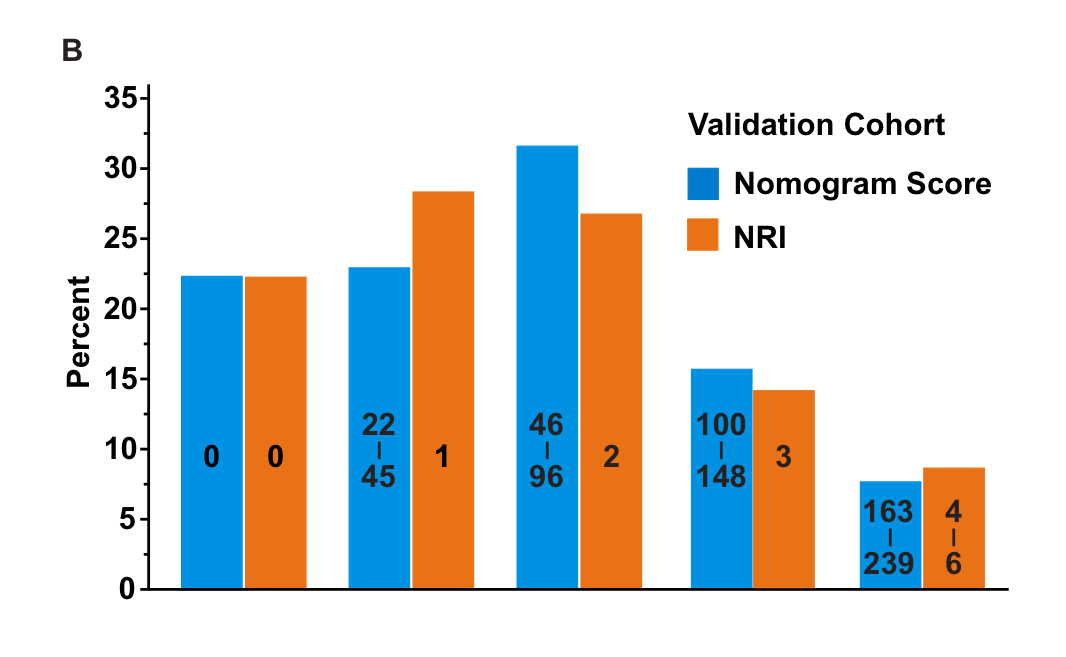

Supplement: Supplementary file 3 — Supplementary Figure 1B [file 41375_2020_791_MOESM3_ESM.tif]

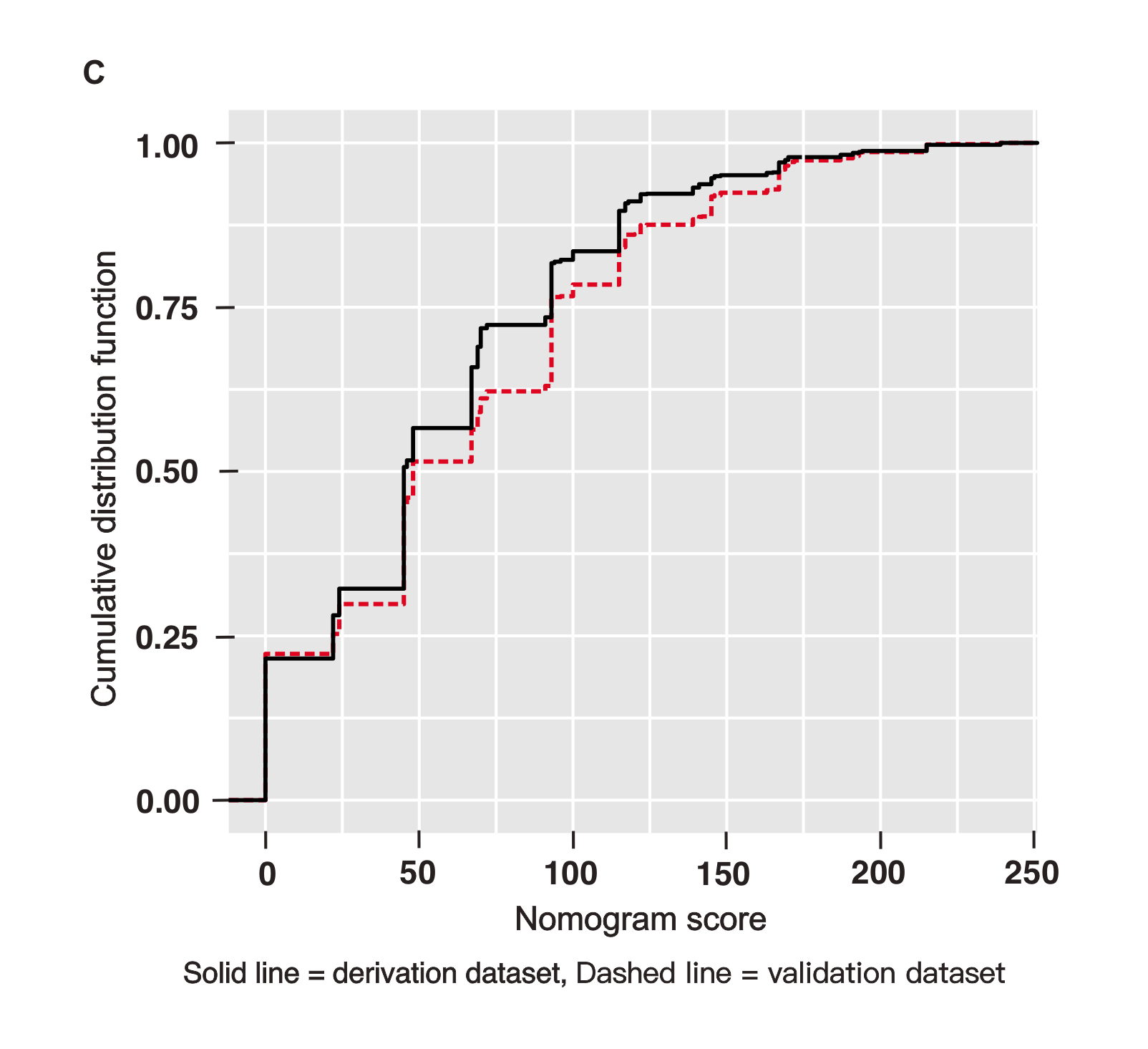

Supplement: Supplementary file 4 — Supplementary Figure 1C [file 41375_2020_791_MOESM4_ESM.tif]

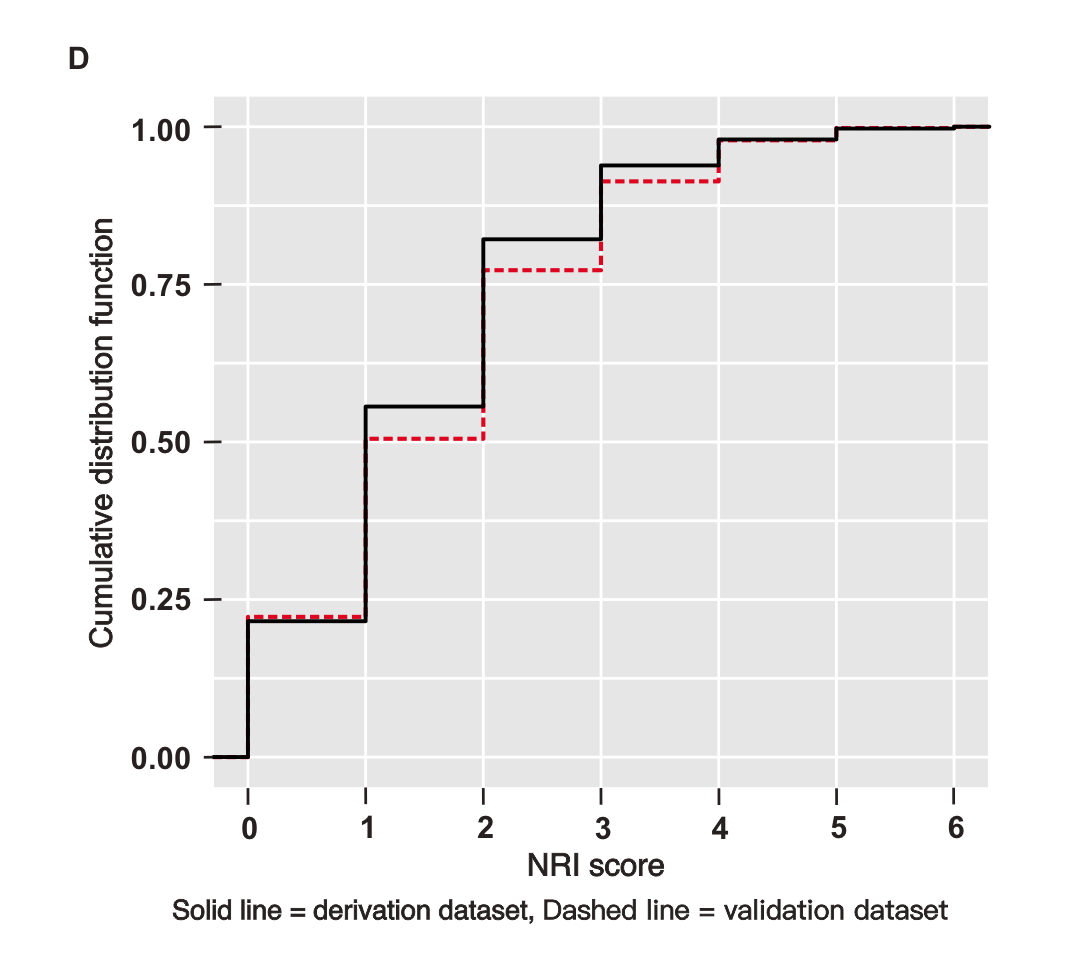

Supplement: Supplementary file 5 — Supplementary Figure 1D [file 41375_2020_791_MOESM5_ESM.tif]

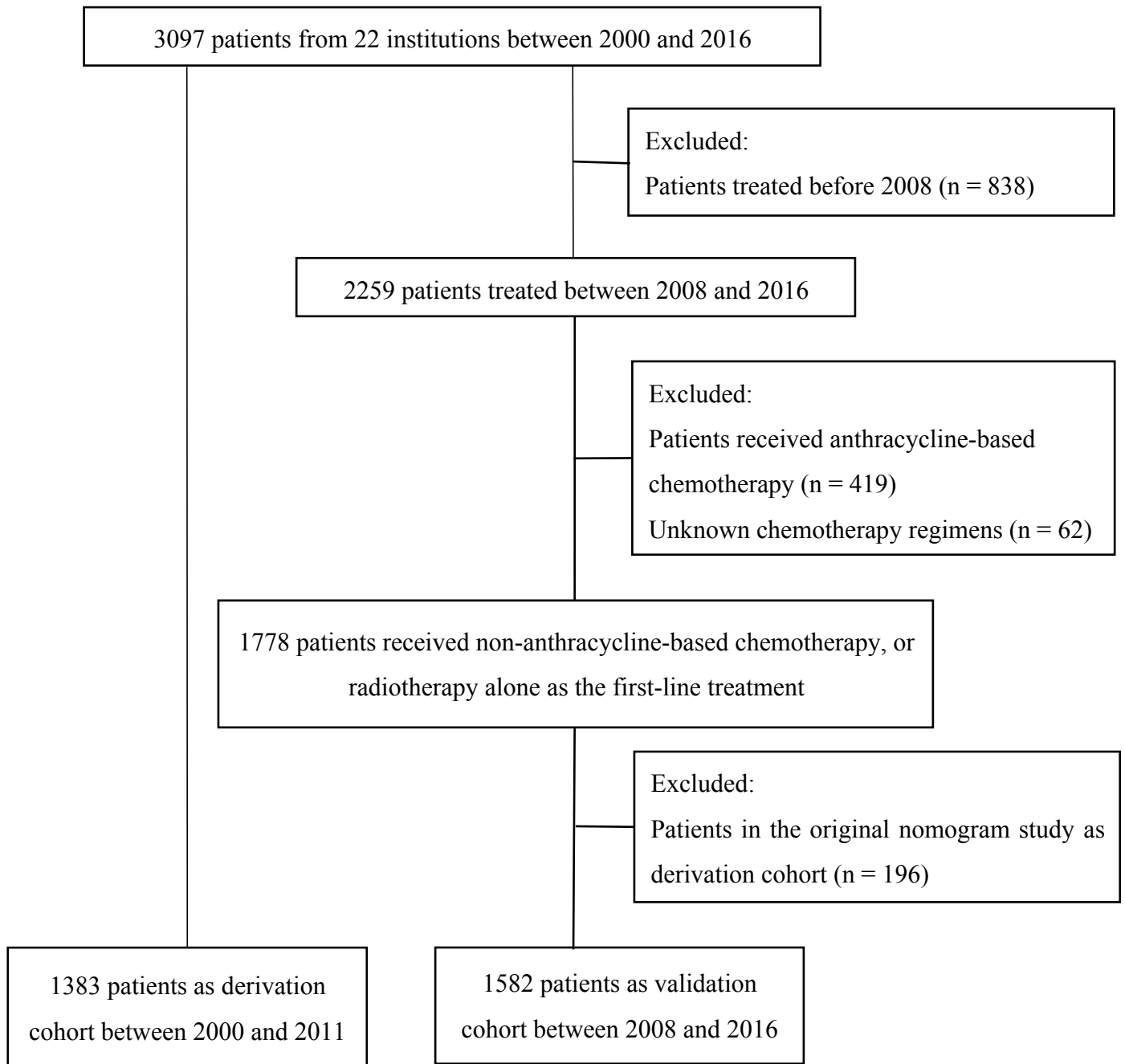

Supplement: Supplementary file 6 — Supplementary Figure 2 [file 41375_2020_791_MOESM6_ESM.pdf]

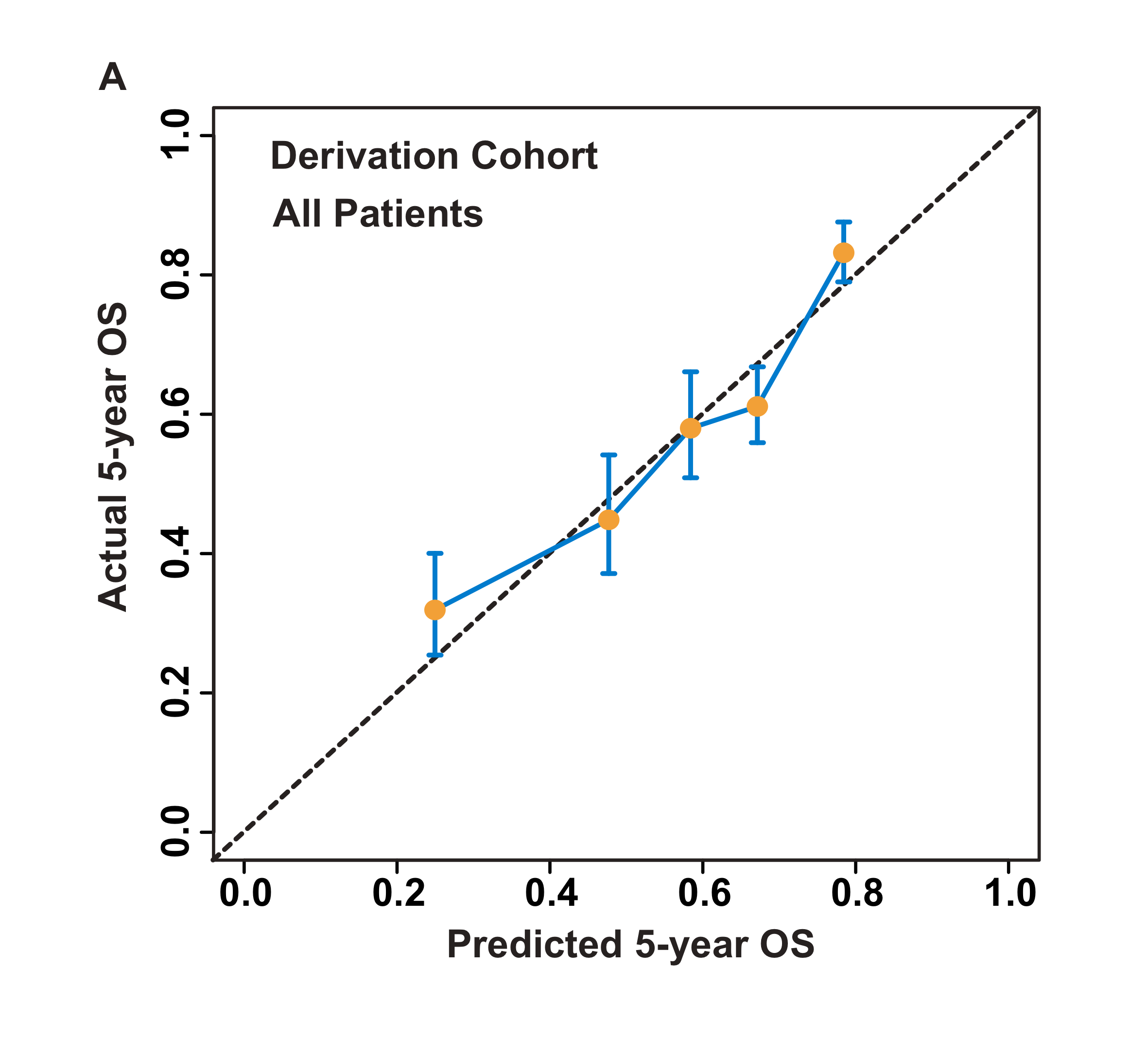

Supplement: Supplementary file 7 — Supplementary Figure 3A [file 41375_2020_791_MOESM7_ESM.tif]

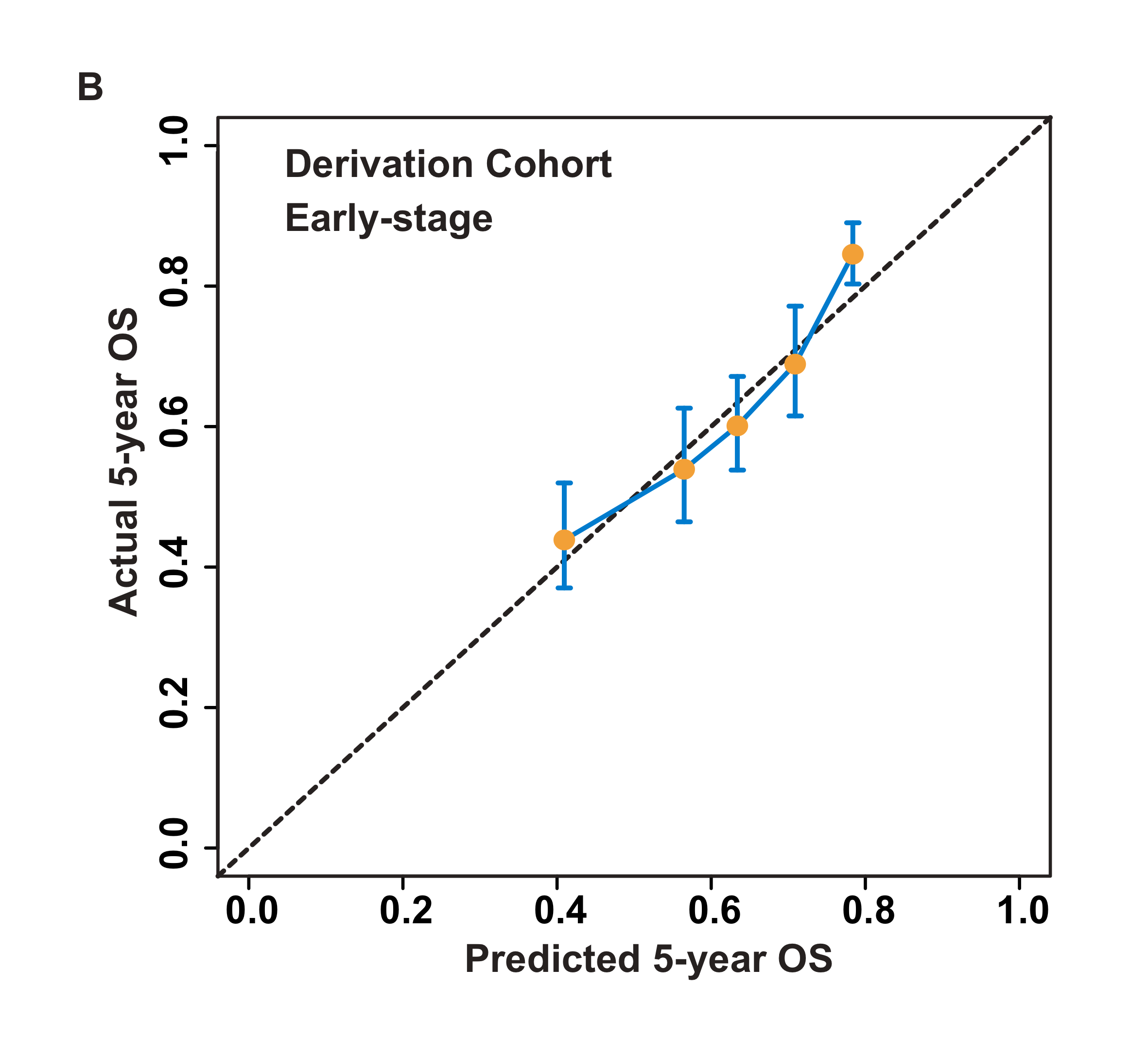

Supplement: Supplementary file 8 — Supplementary Figure 3B [file 41375_2020_791_MOESM8_ESM.tif]

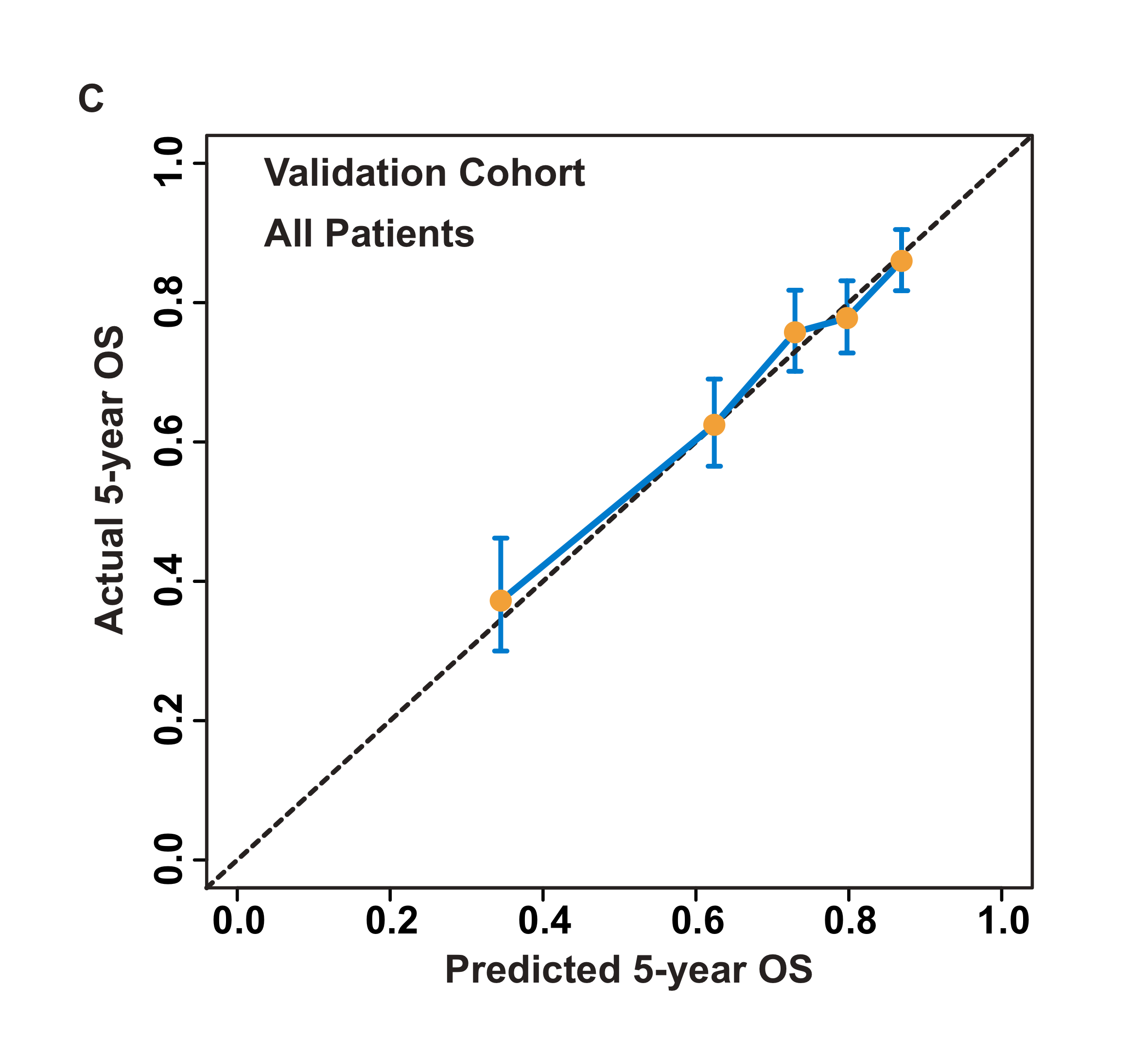

Supplement: Supplementary file 9 — Supplementary Figure 3C [file 41375_2020_791_MOESM9_ESM.tif]

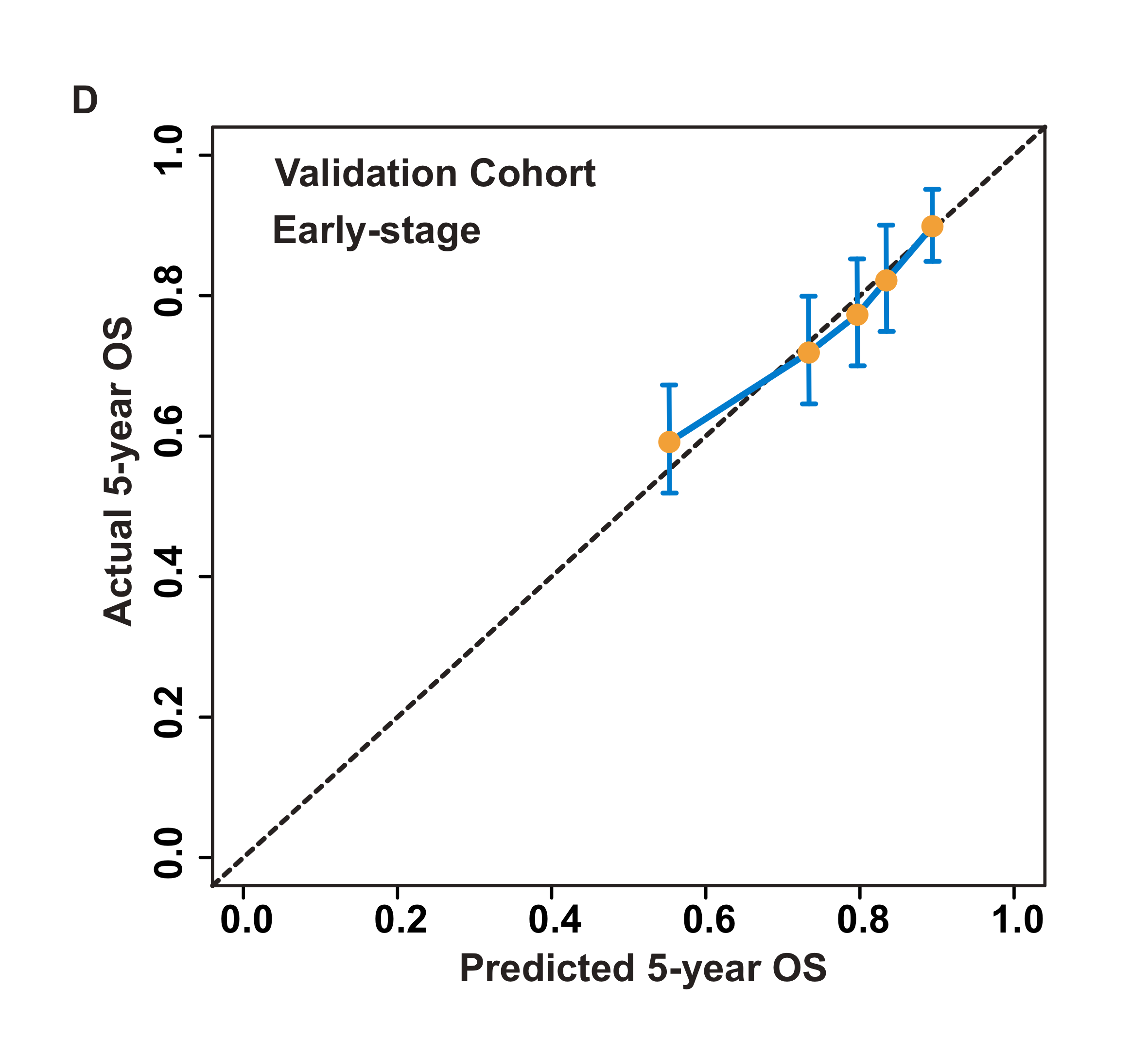

Supplement: Supplementary file 10 — Supplementary Figure 3D [file 41375_2020_791_MOESM10_ESM.tif]

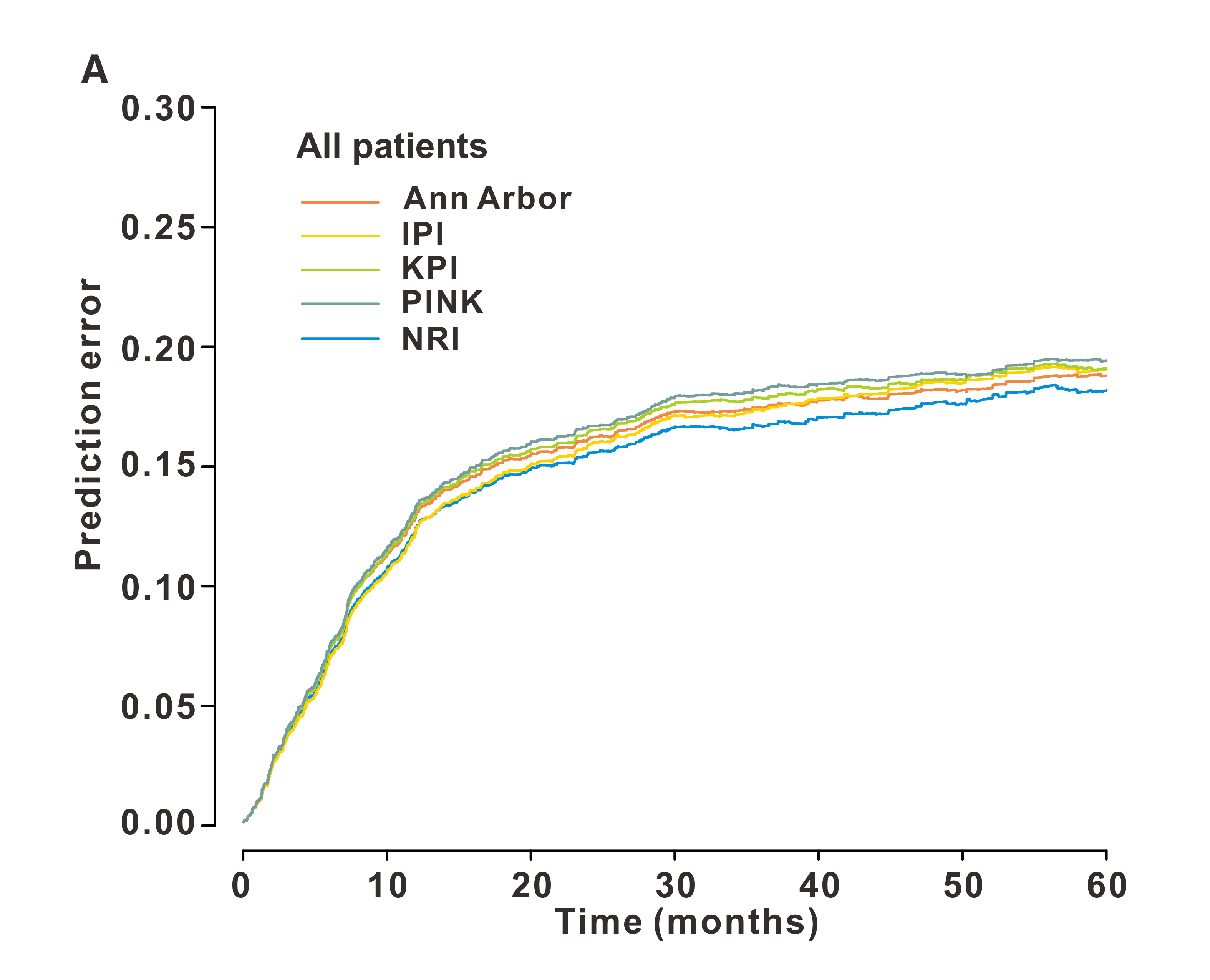

Supplement: Supplementary file 11 — Supplementary Figure 4A [file 41375_2020_791_MOESM11_ESM.tif]

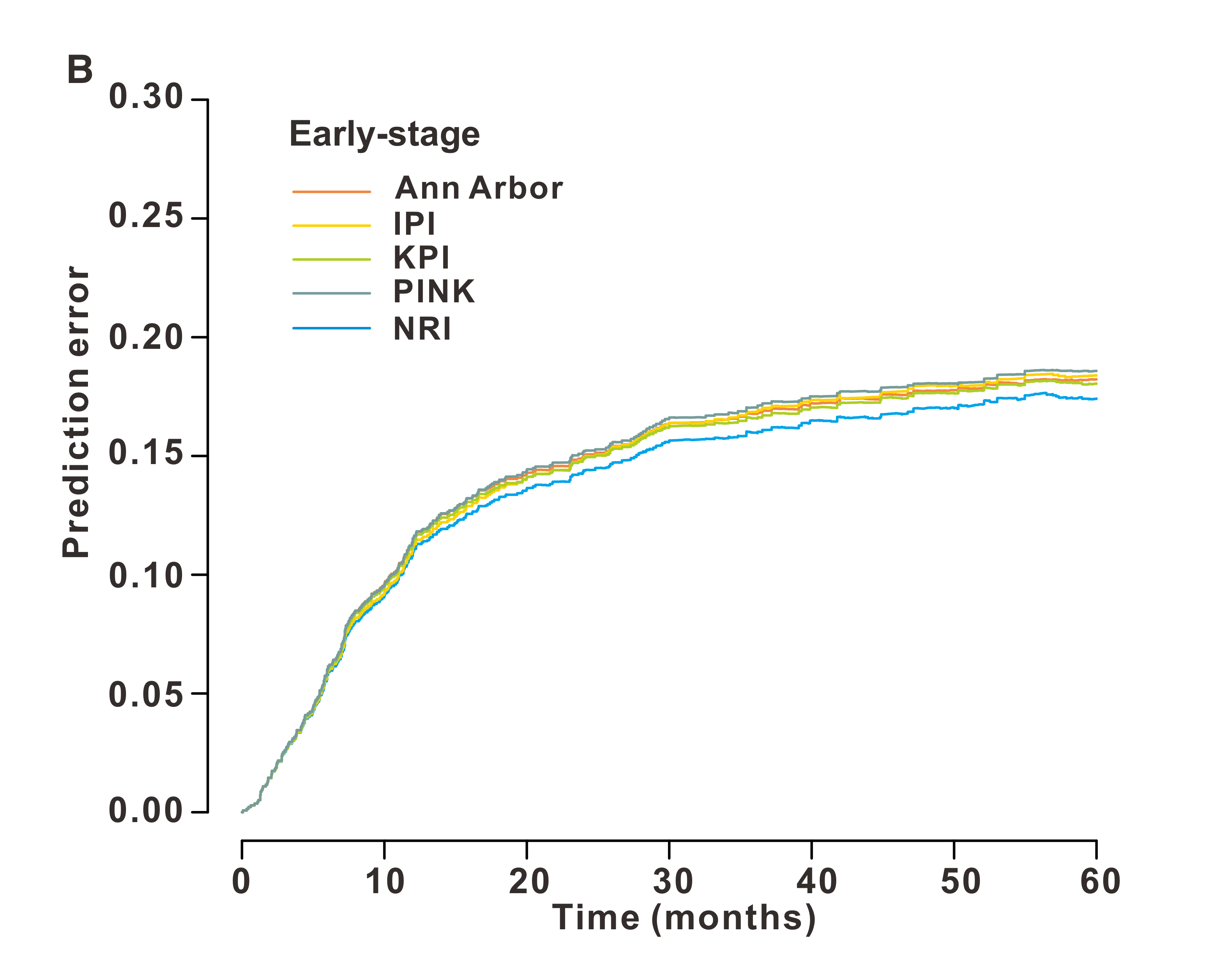

Supplement: Supplementary file 12 — Supplementary Figure 4B [file 41375_2020_791_MOESM12_ESM.tif]
